# COVID-19 outbreak investigations in a school in greater Gaborone, Botswana

**DOI:** 10.11604/pamj.2025.50.110.37551

**Published:** 2025-04-24

**Authors:** Lebapotswe Tlale, Chidzani Catherine Mbenge, Naledi Mokgethi, Katlego Didimalang, Sidney Otlaadisa Kololo, Lesego Releseng, Shaolyn Setabo

**Affiliations:** 1 Health Services Management, Ministry of Health and Wellness, Gaborone, Botswana,; 2 Department of Public Health, University of Botswana, Gaborone, Botswana,; 3 Health Promotion and Education, District Health Management Team, Gaborone, Botswana

**Keywords:** Outbreak investigation, COVID-19, cluster

## Abstract

**Introduction:**

many countries enforced national lockdowns, including the closure of schools, to control the COVID-19 pandemic in 2020. Schools are populated areas, which make them potential spreading grounds for COVID-19 if inadequate infection control and prevention measures are in place. We describe a COVID-19 outbreak in a school that signalled the 1^st^ community transmissions of COVID-19 in Botswana following school resumption.

**Methods:**

a COVID-19 outbreak investigation through contact tracing and assessment of compliance with COVID-19 protocols was conducted at a school in Greater Gaborone after one of the students tested positive.

**Results:**

the index case is a 16-year-old female who had 165 contacts, of which 35.8% (59/165) of the people she was in contact with were possibly exposed and tested positive for COVID-19, while 0.6% (1/165) were lost to follow-up. The private school had 109 students and 8 staff, and a positivity rate of 66/117 (56%). In the index class, 21/25 (84%) students tested positive for COVID-19. Some of the close contacts from this school were linked to 10 other schools.

**Conclusion:**

even though temperature checks are not a reliable screening modality for COVID-19, they led to the detection of community transmissions in Botswana. The reopening of schools is essential for the continuity of education; however, the failure of schools to follow COVID-19 protocols can increase the spread of COVID-19. Before the reopening of schools, control measures should be intensified and we also recommend regular spot checks by authorities to ensure compliance.

## Introduction

COVID-19 is a disease caused by the SARS-CoV-2 virus that first emerged in Wuhan, China, in December 2019 and spread rapidly across the globe, forcing many countries to implement national lockdowns [1]. The World Health Organization declared the pandemic a public health emergency of international concern on the 30^th^ January 2020, looking at its global spread and impact [2]. The pandemic disrupted many sectors, including education, leading to the closure of schools in efforts to contain the virus and reduce mortality [3,4]. The United Nations Educational, Scientific and Cultural Organization (UNESCO) estimated that by March 18, 2020, 107 countries had implemented national school closures related to COVID-19, affecting 862 million children and young people, roughly half the global student population [4].

The closure of schools has negative repercussions as it impacts the continuity of education, and the decisions of when to close or safely reopen schools, which were challenging for policymakers [3,5]. The majority of policymakers were concerned that school reopening would drive community transmissions. Although there was conflicting evidence on the effectiveness of school closures, several studies showed that the reopening of schools had no substantial effects on the reproductive coefficient of SARS-CoV-2 [6-11]. Nevertheless, outbreaks within schools do raise alarms among the young population that is often asymptomatic and has the potential to spread the virus to communities [12-17]. In a Wisconsin school outbreak investigation in the United States, several factors were identified that contributed to transmission of the COVID-19 disease in schools which included: prolonged contact, sharing of quarters and undocumented reports of children [14]. Hence, the reopening of schools should be accompanied by strict infection control and prevention measures within schools, including screening and social distancing among students.

The 1^st^ three imported cases of COVID-19 in Botswana were identified on the 30^th^ March 2020 [18]. Botswana, like many countries, responded swiftly to the COVID-19 pandemic and on the 2^nd^ April, 2020, with only 4 positive cases, His Excellency the President of Botswana declared a State of Public Emergency which curtailed non-essential services and unnecessary movement [19]. This initiated the 1^st^ national lockdown lasting 28 days, including all basic and tertiary schools' closures. The lockdown was further extended to 20^th^ May 2022, allowing a phased approach to easing restrictions [20]. The first phase lasted from 30^th^ April to 7^th^ May, 2020 and the country operated under lockdown restrictions and face masks were mandatory. during phase 2. A trade, business, or school could operate after satisfying the Director of Health Services to prevent the spread of COVID-19 through non-contact temperature screening, disinfection, maintaining COVID-19 registers, social distancing, provision of sanitizers, and use of face masks. Businesses were to operate with 25% staff, then 50% staff. Phase 2 was from the 8^th^ - 20^th^ May, 2020. In Phase 3, which was from 20^th^ May 2022 and beyond, all businesses were to operate at 100% while they adhered to COVID-19 regulations.

During lockdown, the 1^st^ cluster outbreak in the Mogoditshane community described in Mbenge *et al*., 2020, yielded only 8 local transmissions and no further cases were discovered through contact tracing as per national guidelines [21]. On the 20^th^ of May, when restrictions were lifted, Botswana had only 23 confirmed cases of which many were imported cases from travellers and cross-border truck drivers. The schools re-opened on 15^th^ May 2020 for private schools and 2^nd^ June 2020 for government schools after meeting the COVID-19 guidelines set by the Ministry of Health and Wellness and safe school opening guidance from the Ministry of Basic Education [22].

After the relaxation of lockdown restrictions and schools re-opening, COVID-19 cases started to increase, but for a while remained concentrated among cross-border truck drivers until this case investigation. There was routine testing at entry points, but not in the community. We describe an investigation of the first COVID-19 cluster outbreak in schools around Greater Gaborone in Botswana using contact tracing. This outbreak signalled an ongoing community transmission as it led to the identification of clusters of cases across the district and neighbouring districts.

## Methods

**Study design:** we report on a prospective follow-up of a 16-year-old Motswana female who was returned from a private secondary school following a failed screening test with a high temperature test on the 23^rd^ July 2020, 2 months after the re-opening of schools. The case was then tested using PCR COVID-19 at a private hospital on 25^th^ July 2020 and the results came out positive. This triggered a contact investigation as per the Botswana national contact tracing strategy and guidelines.

At the time of dissemination of the results, the index case was home and a contact tracing team was deployed to interview the case and thereafter hand the case to the case management team. The following critical details were established during the interview: the index stays with a family of 8 in a village within the Greater Gaborone Zone; the index case attends private school in a different village about 10 km from home. The parents drop her off at school and she uses public transport to get back from school. Her daily activities at school include attending lessons, interactions with friends, buying snacks from the stalls during tea break and buying lunch at the nearby supermarket; the family owns a local store within the community they live in of which the index case had visited when returning from school; 20^th^ July 2020 - The index reported to have experienced a sore throat, headache and runny nose and she self-isolated at home. She also indicated that four of her classmates had flu-like symptoms, the previous week, 22^nd^ July 2020 - she went to school, where she was returned due to a high temperature of 37.5 degrees Celsius. She used public transport and visited a nearby supermarket where she was not screened and registered. She then passed by the family store before going home; she later visited the Post Office to make some payment where she reported to have washed her hands and was registered by the official. She then went back to the shop; at home she was interacting with other family members, this was also observed during the interview and 23/07/2020- Mother took her for COVID-PCR testing at a private hospital and she was confirmed a case on 25/07/2020. On the same day of 25/07/2020- two of her friends visited her at home and then later she visited three of her friends.

**Participants and study size:** participants included the index case and all the contacts identified during contact tracing. All contacts identified during contact tracing were included in the data analysis.

**Study setting:** the study was conducted in Botswana's Greater Gaborone region and its neighbouring health districts. Botswana is a landlocked country located in Southern Africa [21]. The country neighbours South Africa to the south and west; Namibia to the north and west; Zambia to the north and Zimbabwe to the northeast [21]. The country covers a land area of about 582 000 square kilometres. According to the 2020 Population projections, the country's estimated population is 2,374,698 [20]. Gaborone is the capital city and 11.4% of the country's population lives within the Greater Gaborone area. The country's largest government referral hospital, 3 private hospitals, 30 clinics and 4 health posts are situated in the Greater Gaborone Health District (GGDHMT). The index case lived in Greater Gaborone and attended a small private secondary school within the health district. The school was about 10 km from the Index case's home and has about 117 staff and students.

**Outbreak investigation:** the index case was interviewed and all the people and places she had interacted with were identified. A case investigation was launched comprising a public health specialist, a medical doctor, a general nurse, a health education officer, an environmental health officer, a community health nurse and the cluster contact tracing teams. Contact tracing was carried out for all emerging positive cases from the investigation. All the affected areas and places (Schools, Shops etc.) where COVID-19 cases were identified during the outbreak investigation were assessed for compliance using a standard tool developed by the Presidential Task Team for compliance against the National COVID-19 protocols. The assessment was based on the following: availability of sick/holding bay, social distancing, use of masks, cleaning, temperature screening, and clear risk communication channels.

**Study procedures:** following the index interviews, all cases were handed to case management and isolated at the designated isolation facility- the newly built Sir Ketumile Masire Teaching Hospital. From the contact listing forms, the contact tracing teams called everyone on the list to inform them of their contact status and the need for quarantine. At the time of identification, contacts were followed at their homes and did a spot COVID-19 PCR test and transported to a facility quarantine for 14 days from the last day of exposure to index case, with a 2nd COVID-19 PCR test at day 10. At this phase of the pandemic, both close and casual contacts were quarantined. All contacts were undergoing facility quarantine at outsourced lodges and hotels because of the prior challenges experienced with home quarantine in the country [21].

**Definition of terms**

***Index case:*** the patient in an outbreak who is first noticed by the health authorities, and who makes them aware that an outbreak might be emerging.

***Close contact:*** physical contact or being within 2m of a case for more than 15 minutes, or more than 2 hours in an enclosed space (e.g. household, classroom, office, meeting space, or hospital waiting room, public transport) with a case 4 days before and 14 days after symptoms onset/testing if asymptomatic.

***Casual contact:*** within 2m of a case for less than 15 minutes or less than 2 hours in an enclosed space with the case, 4 days before and 14 days after symptoms onset/testing if asymptomatic.

**Bias:** the possible source of bias could be recall bias when the Index or contacts could not remember their close and casual contact during the investigation. The investigations were done immediately to avoid participants forgetting the important event.

**Variables:** the primary outcome measure includes the number of people exposed and tested positive to COVID-19, transmission pattern and adherence to infection prevention and control measures.

**Data sources:** the standard contact identification and contact listing forms adopted from WHO were used during interviews and contact tracing. Close contacts were tested at day 1 post-exposure and day 10 post-exposure using PCR. A standard IPC standard form was used to assess compliance to IPC measures.

**Statistical methods:** categorical variables were analysed using summary statistics such as frequencies and percentages. For continuous variables such as age, median (inter-quartile range) or mean (and standard deviation) were presented depending on whether the variable under consideration is skewed or not, respectively. A transmission map was developed to identify the possible source of exposure. Tables and Graphs were used for summary statistics and trends respectively.

**Ethical consideration:** this study was conducted according to Botswana, and International Standards of Good Clinical Practice, applicable government regulations and Institutional research policies and procedures. The protocol and any amendments made were submitted to the Botswana, Ministry of Health Institutional Review Board (IRB), where it was approved. The protocol number awarded for this study is HPRD 6/14/1.

## Results

**Participants:** about 35.8% (59/165) of people with possible exposure tested positive for COVID-19 and 0.6% (1/165) were lost to follow-up (Table 1).

**Table 1:** contact tracing indicators for index case

Contact output tracing indicators		Number
Number of People with Possible exposures	Total	165
	Close contacts identified	162
	Casual contacts identified	3
	Transferred out to other districts	0
	Still under investigation for level of risk	0
	Lost to follow up	1
Number of close contacts in facility quarantine		160
Number of close contacts in home quarantine		1
Total number swabbed		161
Not swabbed-lost to follow up		1
Results	Positive	59
	Negative	102
	Pending	0

**Figure 1:**
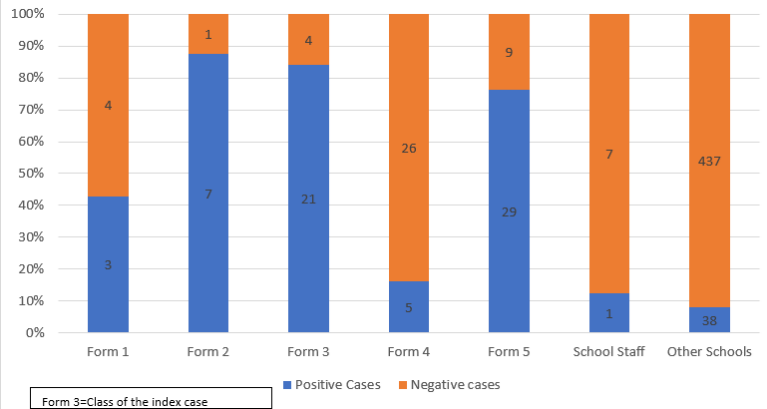
number of cases at index's secondary school

**Descriptive data (school outbreak):** a total of 66/117 (56%) cases tested positive in the Index case's secondary school. In the index class 21/25 84% of the students tested positive for COVID-19 (Figure 1). About 14 students tested positive on second test after previously having tested negative at baseline.

**Involvement of other schools:** a total of 10 schools were involved in the outbreak, yielding 7.7% (38/493) positive cases among close contacts identified (Table 2). A total of 2.9 % (18/613) were lost during follow-up (Table 2). Primary school students recorded the most contacts and junior secondary school recorded the least.

**Table 2:** contacts investigation in schools identified during investigation

No of schools involved in the outbreak	Level of School	Contacts		Number Swabbed	Loss to follow-up	Results	
		Close	Casual			Negative	Positive
5	Primary School	303	56	285	18	254	31
3	Junior Secondary School	16	10	16	0	13	3
2	Senior Secondary School	174	54	174	0	170	4
**10**	**Total**	**493**	**120**	**475**	**18**	**437**	**38**

**Figure 2:**
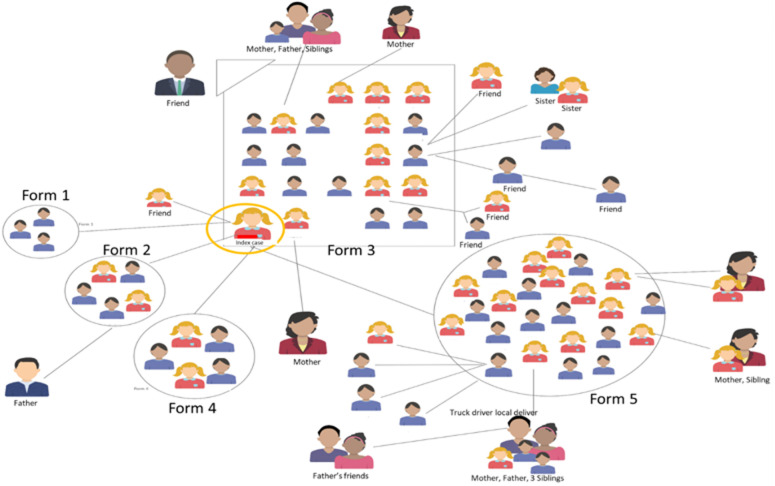
transmission pattern of COVID-19 in Greater Gaborone

**Outcome data (transmission pattern):** Figure 2 shows the transmission pattern identified during contact investigation with the Index case class being the most affected and also members of the community affected in the outbreak.

**Figure 3:**
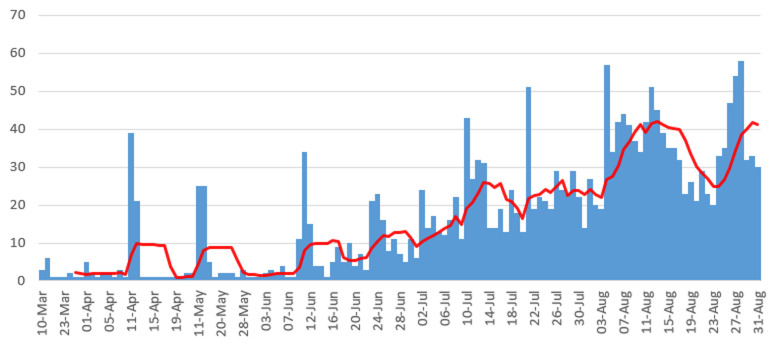
Botswana COVID-19 Epi-curve from March 2020 to August 2020

**Epidemiological curve:** cases started to peak at around the 1^st^ week of July, 2020 and the country started to experience the first COVID-19 wave around August, 2020 as depicted in Figure 3.

**School environment assessment:** the index secondary school assessment showed only temperature screening was properly done and failed in adherence to other COVID-19 protocols (Table 3).

**Table 3:** index school environment assessment

	Compliance		
	Not available	Inadequate	Adequate
Presence of sick bay	X	-	-
Temperature screening	-	-	X
Registration	-	X	-
Use of mask	-	X	-
Social distancing	-	X	-
Cleaning of surfaces (twice daily)	-	X	-
Communication channels	X	-	-

## Discussion

The contact tracing investigation of an index case identified using temperature screening revealed community transmission in Botswana with 92 COVID-19 cases linked to the index case. It also revealed non-adherence to COVID-19 protocols by schools which is critical to reducing the transmission pattern of the disease. This investigation also highlights the importance of contact tracing in identifying cases and reducing transmission of COVID-19 when done effectively.

Botswana opened schools soon after lockdowns and instituted safe school opening measures. Even though in this case the reopening of schools coincided with a peak in local transmission, most studies advice against closure of school to control the epidemic as it does not substantially influence the trajectory of the epidemic [6-11]. The most contributing factor of COVID-19 transmission within the school under investigation was the failure to observe COVID-19 protocols as per findings from the school assessment. These has been observed in other school outbreaks elsewhere [12-17]. Measures that include hand hygiene, social distancing in class, surface disinfections, use of face masks and online classes have been shown to prevent frequent closure of schools and the disruptions of learning when students become close contacts and have to go into quarantine [23,24].

The setting of this investigation was a secondary school of grades 8 to 12 which comprises mainly adolescents. This age bracket has a greater tendency to likely break rules and spread COVID-19 than younger children at primary schools [10-13]. A systematic review and meta-analysis on risk of infection and transmission of SARS- CoV-2 among children and adolescents in educational settings demonstrated that it is largely safe for children (<10 years) to be at schools, while older children (10-19 years) have a higher risk of infection and might facilitate transmission [11]. Additionally, although some studies revealed that majority of cases among students originates from household and community contacts than at schools there remains a concern of transmission within schools [10,16]. In this case investigation both many of the school contacts and household contacts tested positive.

The use on non-contact thermometers for screening was a requirement for entry into any business or school premises in Botswana and the index case was detected using temperature screening alone leading to the identification of the first community transmission occurring in the country. However, studies have revealed temperature screening as a primary screening tool not to be an effective method in identifying cases as majority of cases will be asymptomatic and missed [25,26]. In addition to screening some schools in America implemented periodic testing of students and staff to detect and isolate cases early but this is not always possible in resource limited countries like Botswana [27].

This school outbreak that involved 10 other schools occurred after the easing of lockdown restrictions and resuming business and school operations. Through effective contact tracing and case investigations, a lot of cases were identified involving multiple transmission routes and non-adherence to the country`s COVID-19 protocols. The possible transmission routes in this case were shared school and public transport, shared bathrooms, study groups, social and religious events like weddings and church gatherings. In the UK it was determined that an optimal strategy for reopening schools was to couple school reopening with population-wide testing of symptomatic individuals and effective contact tracing and isolation of cases [28]. This way the virus is contained at community level with few transmissions to the schools.

For the longest Botswana enjoyed good statistics of few local transmissions unbeknown that there was already undetected community transmission until this case investigation. In addition to COVID-19 protocols the country should have put in place measures to detect local/community transmissions as soon as it occurred. Countries like Australia utilised the surveillance plan whereby general practitioners sampled patients with influenza-like illness and tested them for COVID-19 to determine if the virus is circulating in the community undetected [29].

This case investigation is not without limitation as in some instances the contact tracing team conducted investigation without all the disciplines available. This highlights the importance of the Rapid Response Team in outbreak response and element which the Ministry of Health needs to strengthen. A small percentage of contacts were lost to follow-up and could potentially have introduced new pockets of outbreaks as well.

## Conclusion

This case investigation is clear evidence of the effectiveness of contact tracing in detecting and reducing transmissions of COVID-19. It also highlights the importance of adhering to COVID-19 protocols to control the disease especially in educational settings to keep schools open. The Ministry of Health is recommended to have a surveillance plan for influenza-like illnesses for early detection of outbreaks. The Ministry of Health should also capacitate the District Rapid Response Teams to respond effectively to future outbreaks and conduct routine inspections of schools and businesses to ensure compliance.

### What is known about this topic

- Contact tracing and contact investigation is known to be an important aspect of active surveillance to early identify and isolate cases to reduce transmission and implement early treatment to reduce mortality;
- Temperature checks/screening are not a good screening tool for COVID-19 disease.

### What this study adds

- This study provides knowledge and when implemented properly, can help establish transmission patterns and come up with ways of closing gaps during a pandemic to reduce further transmission;
- It equally highlights the importance of funding active surveillance activities to reduce the socio-economic impact of COVID-19 disease;
- The study also shows that using skilled personnel for screening is essential to properly manage COVID-19/influenza to reduce the spread of transmission.
